# TRIM25 Enhances the Antiviral Action of Zinc-Finger Antiviral Protein (ZAP)

**DOI:** 10.1371/journal.ppat.1006145

**Published:** 2017-01-06

**Authors:** Melody M. H. Li, Zerlina Lau, Pamela Cheung, Eduardo G. Aguilar, William M. Schneider, Leonia Bozzacco, Henrik Molina, Eugen Buehler, Akinori Takaoka, Charles M. Rice, Dan P. Felsenfeld, Margaret R. MacDonald

**Affiliations:** 1 Laboratory of Virology and Infectious Disease, The Rockefeller University, New York, New York, United States of America; 2 Integrated Screening Core, Experimental Therapeutics Institute, Icahn School of Medicine at Mount Sinai, New York, New York, United States of America; 3 Proteomics Resource Center, The Rockefeller University, New York, New York, United States of America; 4 Trans-NIH RNAi Screening Facility, Division of Preclinical Innovation, National Center for Advancing Translational Sciences, NIH, Rockville, Maryland, United States of America; 5 Division of Signaling in Cancer and Immunology, Institute for Genetic Medicine, Hokkaido University, Sapporo, Japan; Icahn School of Medicine at Mount Sinai, UNITED STATES

## Abstract

The host factor and interferon (IFN)-stimulated gene (ISG) product, zinc-finger antiviral protein (ZAP), inhibits a number of diverse viruses by usurping and intersecting with multiple cellular pathways. To elucidate its antiviral mechanism, we perform a loss-of-function genome-wide RNAi screen to identify cellular cofactors required for ZAP antiviral activity against the prototype alphavirus, Sindbis virus (SINV). In order to exclude off-target effects, we carry out stringent confirmatory assays to verify the top hits. Important ZAP-liaising partners identified include proteins involved in membrane ion permeability, type I IFN signaling, and post-translational protein modification. The factor contributing most to the antiviral function of ZAP is TRIM25, an E3 ubiquitin and ISG15 ligase. We demonstrate here that TRIM25 interacts with ZAP through the SPRY domain, and TRIM25 mutants lacking the RING or coiled coil domain fail to stimulate ZAP’s antiviral activity, suggesting that both TRIM25 ligase activity and its ability to form oligomers are critical for its cofactor function. TRIM25 increases the modification of both the short and long ZAP isoforms by K48- and K63-linked polyubiquitin, although ubiquitination of ZAP does not directly affect its antiviral activity. However, TRIM25 is critical for ZAP’s ability to inhibit translation of the incoming SINV genome. Taken together, these data uncover TRIM25 as a bona fide ZAP cofactor that leads to increased ZAP modification enhancing its translational inhibition activity.

## Author Summary

Organisms have evolved various innate strategies to defend against pathogens. During virus infection, the cell senses the viral nucleic acid and produces type I interferon, which alerts the neighboring cells. Signaling of type I interferon triggers expression of a wide array of genes, some of which encode proteins that inhibit the replication of diverse virus families. It is not clear what determines the broad yet specific activity of these interferon-induced gene products. Identification of cellular cofactors and pathways required for the function of these broadly antiviral proteins would help elucidate their mechanisms. Here, we perform a genome wide knockdown screen to identify host factors important for the antiviral action of zinc finger antiviral protein (ZAP), a broad-spectrum inhibitory protein induced by interferon. We find that ZAP synergizes with host proteins with divergent functions and identify TRIM25 as a bona fide ZAP cofactor. TRIM25 binds to both splice isoforms of ZAP, and stimulates their ubiquitination and function by facilitating the ability of ZAP to block viral translation. Our data sheds light on the antiviral mechanism of ZAP and advances our understanding of host factor contributions to innate immune responses against viral infections.

## Introduction

The recent re-emergence and spread of viruses beyond their normal geographic distribution have affected countries worldwide. Understanding the biology of host factors with broad antiviral activity is crucial to vaccine and drug development efforts to counteract existing and emerging viral infections.

As a first line of defense against viruses, the host produces type I interferons (IFNs), which signal through the JAK/STAT pathway to induce hundreds of IFN-stimulated genes (ISGs) that block various steps of the viral life cycle (reviewed in [1]). Among the ISGs, zinc finger antiviral protein (ZAP), encoded by the *ZC3HAV1* gene, inhibits alphaviruses, filoviruses, hepatitis B virus, retroviruses, and the LINE-1 and Alu retroelements [2–8]. However, ZAP does not inhibit yellow fever virus, vesicular stomatitis virus, and herpes simplex virus 1 (HSV-1) [3]. It is not well understood what determines the broad yet specific antiviral activity of ZAP.

ZAP, also called PARP13, is a member of the poly(ADP-ribose) polymerase (PARP) family and is alternatively spliced. The long isoform of ZAP (ZAPL) contains a PARP-like domain on the C-terminus that is missing in the short isoform (ZAPS). This PARP-like domain is not enzymatically active [9], although exchange of the inactive catalytic triad in ZAPL to that of the active PARPs completely abolishes its antiviral activity [10], suggesting an important yet unknown role of the PARP-like domain in the antiviral function of ZAP. Several studies have demonstrated distinct activities for the two isoforms. ZAPL is more active against alphaviruses, such as SINV and Semliki Forest virus, than ZAPS, and carries signatures of positive selection [11, 12]. While both isoforms are induced by IFN, ZAPS is upregulated more than ZAPL by virus infection and type I IFN [5, 13, 14].

Diverse cellular pathways have been implicated in ZAP’s function (reviewed in [15]), but its precise mechanism is unknown. It is possible that ZAP interacts with multiple host factors, and the involvement of those factors in the viral life cycle is what provides the specificity. For example, ZAP binds RNA and recruits the exosome complex to target viral RNAs for degradation [5–7, 16–18]. ZAP also directly inhibits translation of the incoming alphaviral genome [3], with interference in the interaction between eIF4A and eIF4G [19] implicated as one mechanism. In addition, ZAP synergizes with other ISGs for its maximal activity and upregulates RIG-I-mediated IFN-β production [14, 20]. These studies support a model in which ZAP interacts with various host factors and cellular complexes to achieve an optimal antiviral state against diverse viruses.

In an attempt to unify the divergent pathways in which ZAP is involved and to uncover novel cofactors that are important for ZAP’s inhibitory activity, we performed a genome-wide siRNA screen in a cell line inducible for ZAP expression. Large-scale RNAi screens allow us to take an unbiased approach to interrogate every gene in the genome. However, off-target effects lead to false positive hits and severely limit the value of genome-wide screens [21, 22]. To address this we performed a rigorous set of confirmatory assays to verify the top hits and exclude off-target effects. We identified several genes that synergize with ZAP to target SINV or inhibit SINV independently of ZAP. Among the hits, TRIM25 was validated to be a cofactor of ZAP. TRIM25 is an E3 ubiquitin and ISG15 ligase, and is responsible for the polyubiquitination and activation of RIG-I [23–25]. We generated CRISPR clones in *ZC3HAV1*-knockout 293T cells where TRIM25 expression is significantly reduced and further confirmed that TRIM25 synergizes with both ZAPS and ZAPL to block SINV replication. Our data demonstrates that TRIM25 triggers ubiquitination of ZAP and enhances its antiviral activity through inhibition of viral translation, highlighting the importance of cofactors in the mechanisms of broadly antiviral proteins.

## Results

### A genome-wide siRNA screen reveals novel host factors that synergize with ZAP or exhibit ZAP-independent antiviral activity

**Fig 1:**
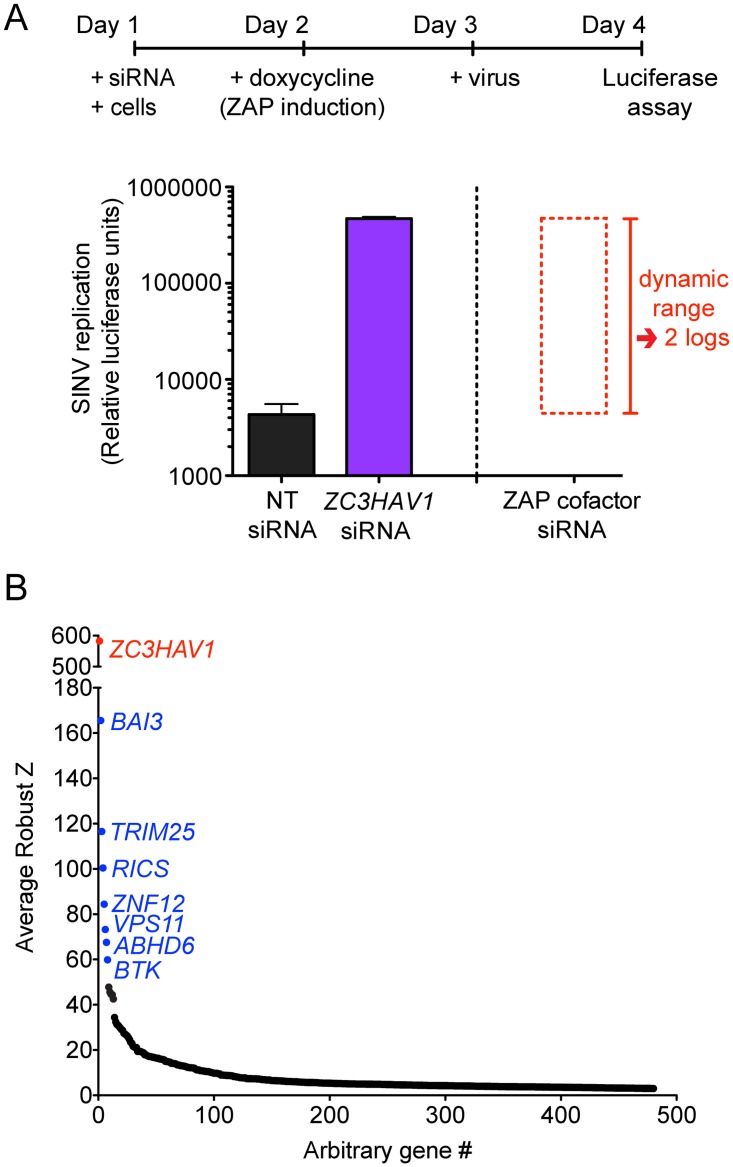
A loss-of-function RNAi screen uncovers many genes that significantly reduce the antiviral activity of ZAP when silenced. **(A)** The experimental outline of the genome-wide siRNA screen is shown. T-REx-hZAP cells transfected with control or gene-specific siRNA were treated with doxycycline to induce ZAPS overexpression one day post-transfection and infected with SINV Toto1101/Luc two days post-transfection. Cell lysates were harvested for measurement of luciferase activity at 24 h post-infection (p.i.). Relative luciferase units represent the level of SINV replication. Cells treated with the control non-targeting (NT) pooled siRNA have low SINV replication while ZAP knockdown by *ZC3HAV1*-specific pooled siRNA rescues viral replication by 2 logs. The large dynamic range in which hypothetical hits (ZAP cofactors) were identified is plotted on the right side of the graph. **(B)** Pooled siRNAs targeting the entire human genome (Dharmacon) were tested in triplicate and genes with an average robust Z score of greater than 3 are plotted. *ZC3HAV1* is highlighted in red while the top hits immediately following *ZC3HAV1* are highlighted in blue.

We performed a genome-wide screen with pooled siRNAs from Dharmacon to identify genes that are required for ZAP’s antiviral activity (see Fig 1A for screen workflow). Viral replication is low in T-REx-hZAP cells when ZAP expression is induced, and silencing of *ZC3HAV1* increases replication of a luciferase-encoding SINV, Toto1101/Luc, by 2 logs. The premise of the screen is as follows: should knockdown of an essential cofactor render ZAP nonfunctional, viral replication will be restored, resulting in increased luciferase activity (refer to “ZAP cofactor siRNA” column in Fig 1A). The screen was performed in triplicate to improve robustness, and we identified 480 genes, whose silencing significantly elevated SINV Toto1101/Luc replication with an average robust Z score of >3 (Fig 1B). As expected, *ZC3HAV1* was the top hit with an average robust Z score of 582.65; this was followed by *BAI3* (165.56), *TRIM25* (116.52) and *RICS* (100.42) (see S1 Table for the entire results).

Normalized percent activation (NPA) and robust Z score were utilized for hit selection [26]. The genes with the highest NPA and robust Z score >3 in all three replicate wells were chosen for validation in a secondary screen (91 genes). Since siRNAs can act as microRNAs and target genes non-specifically through their seed sequences [27], we included 4 genes that were potential off-target candidates from Haystack analysis (*PDIK1L*, *SNAP25*, *FOXK1*, *DGAT2L3*). In addition, we included 1 gene based on overlap with ISGs that we previously found as synergistic with ZAP in an overexpression screen (*MAP3K14*; [20]), and 6 genes from pathways that were significantly enriched but were not on the top 91 list (*APC*, *FZD2*, *GFRA1*, *JAK1*, *SP1*, and *WNT8B*).

**Fig 2:**
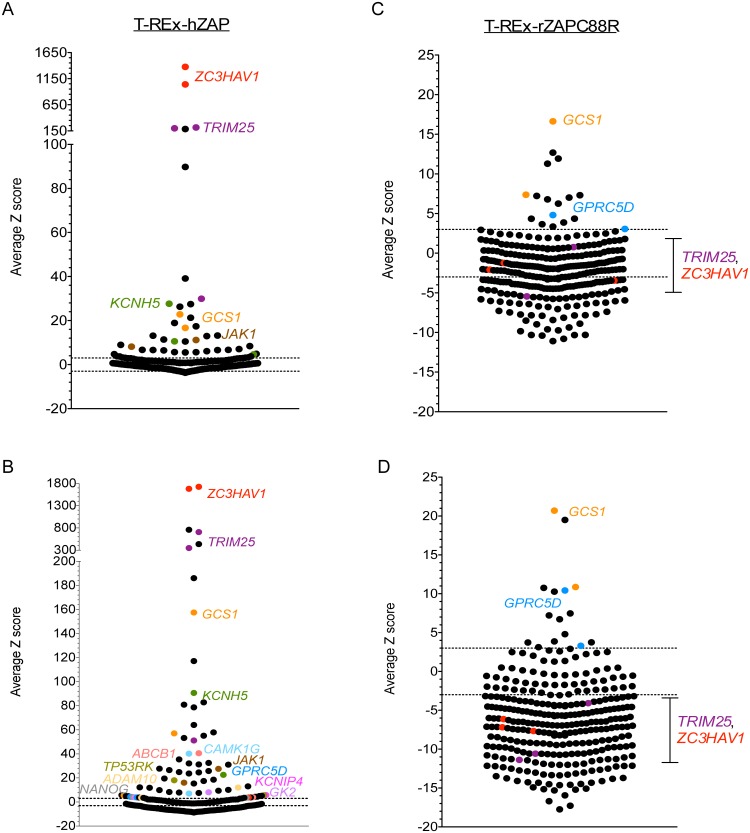
Secondary screen confirms the ZAP-dependent and -independent antiviral effects of hits. A customized library of individual siRNAs (Ambion) targeting 102 hits from the primary screen was tested in triplicate in two different cell lines. Distribution of average Z scores for each of the 3 siRNAs targeting a candidate gene in the secondary screen is shown here. The screen was performed with **(A)** 6.25 nM or **(B)** 25 nM individual siRNAs in 293 cells induced to express ZAPS (T-REx-hZAP), and with **(C)** 6.25 nM or **(D)** 25 nM individual siRNAs in 293 cells induced to express the rZAPC88R dominant negative mutant (T-REx-rZAPC88R). Each dot represents the average Z score of an individual siRNA tested in triplicate. Silenced genes with an average Z score of >3 for at least 2 out of 3 siRNAs are identified and the siRNAs are labeled in color. **(C and D)** The average Z scores of *TRIM25* and *ZC3HAV1* are also plotted to indicate that TRIM25 does not have ZAP-independent antiviral effects.

We re-screened the candidate genes with a library of single siRNAs obtained from a different company (Ambion) to exclude hits that are mediated by off-target effects from further characterization. Knockdown of 5 genes by 6.25 nM siRNA (Fig 2A) and knockdown of 13 genes by 25 nM siRNA (Fig 2B) significantly increased SINV Toto1101/Luc replication (see S2 Table for the entire results). Among them, *ZC3HAV1* was identified as the top hit. *TRIM25*, *KCNH5*, *GCS1* and *JAK1* were also hits at both siRNA concentrations. In addition to the T-REx-hZAP cells used for the primary screen, a 293 clone that is inducible for the expression of rat ZAP C88R mutant, a dominant negative inhibitor of ZAP function [28], was also tested in parallel. Since endogenously expressed ZAP is antiviral, this cell line inhibited for ZAP function allowed us to identify hits with a ZAP-independent antiviral role. Silencing of *GCS1* and *GPRC5D* by 6.25 nM (Fig 2C) and 25 nM (Fig 2D) siRNAs significantly increased SINV Toto1101/Luc replication in these cells where ZAP is not functional (see S2 Table for the entire results), suggesting that they might inhibit SINV in a ZAP-independent manner.

### TRIM25 is a bona fide cofactor of ZAP

**Fig 3:**
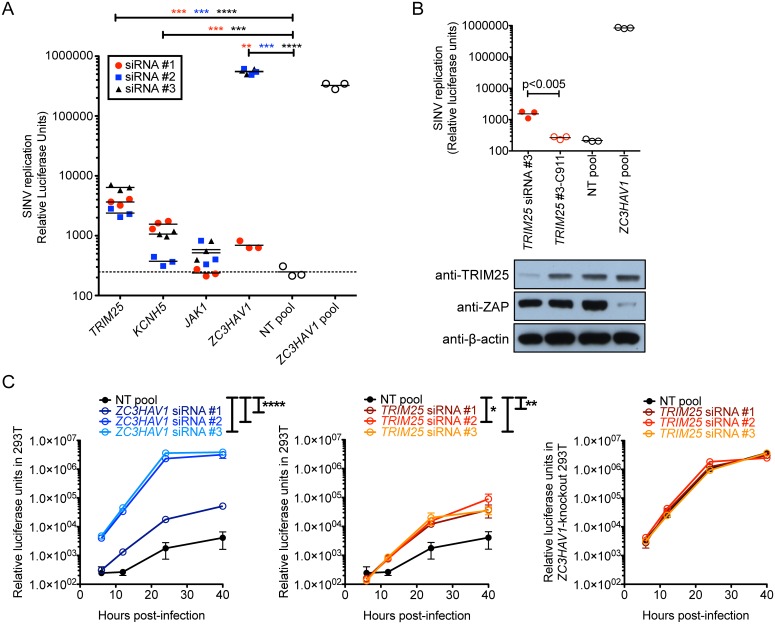
TRIM25 synergizes with ZAP to block SINV replication. **(A)** Candidate genes that significantly increased SINV replication in the secondary screen when silenced by individual siRNAs at both concentrations were validated in a larger scale 24-well plate format. Triplicate wells of T-REx-hZAP cells were transfected with the indicated siRNA, induced to express ZAPS, and infected with SINV Toto1101/Luc at a MOI of 10. Each symbol represents the value obtained from a single well after 24 h of infection. White circles represent results using pooled siRNA controls that were either NT or *ZC3HAV1*-specific. The data is representative of 3 independent experiments. Asterisks indicate statistically significant differences (Student’s t-test, **, p<0.005; ***, p<0.0005; ****, p<0.0001). **(B)** Triplicate wells of T-REx-hZAP cells were transfected with the indicated siRNA, induced to express ZAPS, and infected with SINV Toto1101/Luc at a MOI of 10. Protein expression levels of TRIM25 and ZAP for the same transfections in a duplicate well were determined by immunoblotting. β-actin was used as a loading control. The data is representative of 4 independent experiments. The p-value from Student’s t-test is shown. **(C)** SINV replication in infected 293T cells in which *ZC3HAV1* (left) or *TRIM25* (middle) were silenced, and in *ZC3HAV1*-null 293T cells in which *TRIM25* was silenced (right) is plotted. At 48 h post-transfection with siRNA, cells were infected with SINV Toto1101/Luc at a MOI of 0.01, and lysed at 6, 12, 24, and 40 h p.i. for measurement of luciferase activity. The data is representative of 3 independent experiments. Asterisks indicate statistically significant differences (two-way ANOVA, *, p<0.05; **, p<0.01; ****, p<0.0001).

Next, we validated the candidate ZAP cofactors in a lower throughput assay. Silencing of *TRIM25*, *KCNH5*, *JAK1*, and *ZC3HAV1* led to increased virus replication for at least 2 of the 3 siRNAs (Fig 3A), which was consistent with reduced protein levels of TRIM25 (S1A Fig) and ZAP (S1B Fig), and reduced mRNA levels of KCNH5 and JAK1 (S1C Fig). Among the candidates, 3 independent siRNAs targeting *TRIM25* significantly increased SINV Toto1101/Luc replication by 10- to 26-fold compared to the NT control (Fig 3A). Since *TRIM25*-targeting siRNA #3 was most efficient at rescuing SINV Toto1101/Luc replication, we designed and tested an additional C911 control. The 9^th^ to 11^th^ nucleotides of the siRNA were mutated to their complementary sequence, hence the designation C911, to rule out the possibility that the knockdown phenotype was due to the off-target effects of the siRNA [29, 30]. The C911 control should lose its siRNA activity but still maintain its off-target effects as a microRNA. While *TRIM25*-targeting siRNA #3 rescued SINV Toto1101/Luc replication by about 1 log compared to the NT control, #3-C911 did not lead to increased viral replication and did not reduce the protein level of TRIM25 (Fig 3B), suggesting that the phenotype of siRNA #3 is due to specific silencing of *TRIM25* and not inhibition of an off-target gene. Furthermore, *TRIM25* was silenced in *ZC3HAV1*-knockout 293T cells [14], which were then infected by SINV Toto1101/Luc to determine whether endogenously expressed TRIM25 has a ZAP-independent antiviral role. As controls, *ZC3HAV1* and *TRIM25* were silenced in 293T cells. While *ZC3HAV1* (Fig 3C; left) and *TRIM25* (Fig 3C; middle) silencing in 293T cells restored SINV Toto1101/Luc replication by 1–3 logs by 40 h p.i., silencing of *TRIM25* in *ZC3HAV1*-knockout cells did not further increase SINV Toto1101/Luc replication compared to the NT control (Fig 3C; right). These data suggest that TRIM25 requires ZAP for its anti-SINV activity.

### TRIM25 interacts with ZAP through its SPRY domain

**Fig 4:**
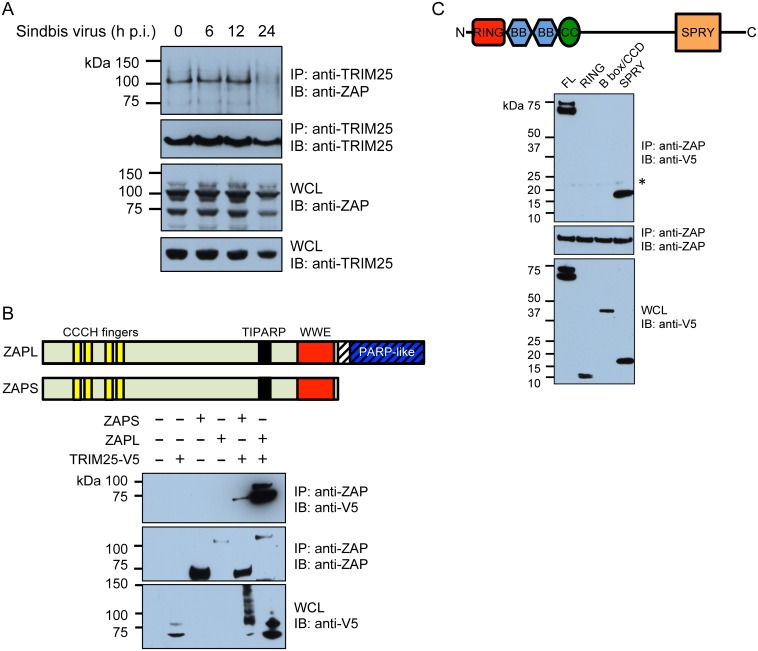
The SPRY domain of TRIM25 associates with ZAP. **(A)** 293T cells were infected with the SINV strain Toto1101 (MOI = 10), and lysates were harvested at 0, 6, 12 and 24 h p.i. for co-immunoprecipitation with an anti-TRIM25 antibody and immunoblotting. The data is representative of 2 independent experiments. **(B)** Human ZAP short (S) and long (L) isoforms are shown, with the CCCH fingers, TIPARP, WWE and PARP-like domains indicated. Light green shading indicates sequences shared by the two isoforms whereas the hatched region containing the PARP-like domain is unique to the L isoform. WCL of *ZC3HAV1*-knockout 293T cells transfected with V5-tagged TRIM25 and/or ZAPS or ZAPL were used for co-immunoprecipitation with an anti-NZAP antibody and immunoblotting. **(C)** Full-length (FL) TRIM25 is shown, with the RING, B box, CCD, and SPRY domains indicated. WCL of *ZC3HAV1*-knockout 293T cells transfected with V5-tagged FL or truncated TRIM25 (RING, B box/CCD, SPRY only) and ZAPL were used for co-immunoprecipitation with an anti-NZAP antibody and immunoblotting. Different amounts of TRIM25 domain-expressing constructs were used for transfection in order to achieve similar RING, B box/CCD, and SPRY expression. The asterisk indicates a non-specific band. **(B and C)** The data is representative of 3 independent experiments.

Next, we asked whether TRIM25 physically interacts with ZAP. We infected 293T with endogenous TRIM25 and ZAP expression with SINV, and immunoprecipitated TRIM25 to look for ZAP association at various time points following infection. We found that endogenous ZAP co-immunoprecipitated with endogenous TRIM25 over the course of SINV infection (Fig 4A). There were less TRIM25 and ZAP proteins present at 24 h p.i., which is consistent with the cytopathic effects observed at that time point, leading to less TRIM25 and ZAP pulldown. Previously, TRIM25 was found to interact with ZAP in the presence of RNA although only ZAPL was investigated [8]. To determine the interaction of TRIM25 and different ZAP isoforms, ZAPS or ZAPL, and/or TRIM25 were co-expressed in *ZC3HAV1*-knockout 293T cells, which were harvested for co-immunoprecipitation. Immunoprecipitation of both ZAPS and ZAPL by a monoclonal antibody recognizing the N-terminal portion of human ZAP (NZAP) pulled down TRIM25, although more TRIM25 is associated with ZAPL (Fig 4B). TRIM25 was dramatically modified in the presence of ZAPS, evident by the presence in the whole cell lysates (WCL) of a ladder of bands larger than the molecular weight of TRIM25 (Fig 4B; WCL). TRIM25 consists of a RING domain, two B box domains, a coiled coil (CCD) domain, and a SPRY domain. The RING domain encodes the ubiquitin ligase activity while the CCD domain is required for oligomerization of TRIM proteins and the SPRY domain is important for mediating protein interactions and substrate specificity [31]. Next, we asked which domain in TRIM25 was responsible for interaction with ZAP. Since more ZAPL associates with TRIM25, we co-expressed similar levels of individual TRIM25 domains with ZAPL and immunoprecipitated ZAPL. We found that the SPRY domain of TRIM25 but not its RING and B box/CCD domains co-immunoprecipitated with ZAPL (Fig 4C). These data suggest that both ZAP isoforms form a complex with TRIM25, likely through interaction with the SPRY domain of TRIM25.

### Both the E3 ubiquitin ligase activity and oligomerization of TRIM25 are required for its functional interaction with ZAP

**Fig 5:**
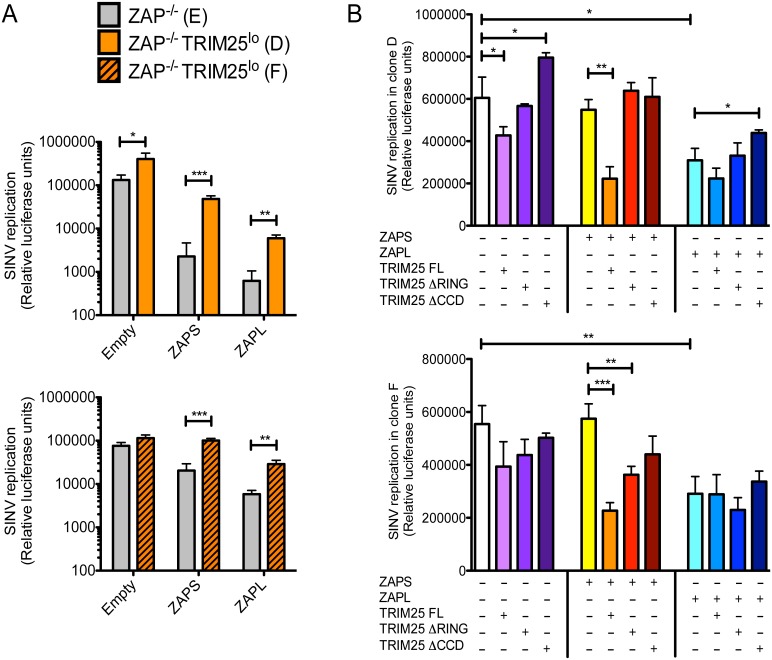
CRISPR targeting of *TRIM25* leads to increased virus replication and both the RING and CCD domains of TRIM25 are required for ZAP activation. **(A)** Wild type (clone E) and TRIM25^lo^ *ZC3HAV1*-knockout 293T cells (clones D and F) were transfected with empty vector or vector expressing ZAPS or ZAPL and infected with Toto1101/Luc (MOI = 0.01) 2 days post-transfection. **(B)** TRIM25^lo^ *ZC3HAV1*-knockout 293T cells (clones D and F) were reconstituted with expression of FL or truncated TRIM25 (ΔRING, ΔCCD) and/or ZAPS or ZAPL, and infected with Toto1101/Luc (MOI = 10) 2 days post-transfection. **(A and B)** The data is representative of 2 independent experiments performed on both clones D and F. Cell lysates were harvested for measurement of luciferase activity at 24 h p.i. Relative luciferase units represent the level of SINV replication. Asterisks indicate statistically significant differences (Student’s t-test, *, p<0.05; **, p<0.005; ***, p<0.0005).

Since TRIM25 is an E3 ubiquitin ligase and has been shown to be important for ubiquitinating RIG-I and upregulating RIG-I-mediated IFN-β production, we hypothesized that TRIM25 also ubiquitinates ZAP and/or other proteins that complex with ZAP in order to stimulate ZAP’s antiviral activity. To test this, we targeted *TRIM25* by CRISPR in *ZC3HAV1*-knockout 293T cells to interrogate the functional interaction between ZAP isoforms and TRIM25. CRISPR targeting led to either in-frame deletions in all three chromosomal copies of *TRIM25* (clone D) or frameshift insertions in two chromosomal copies and an in-frame deletion in one (clone F), consistent with the almost undetectable protein expression of TRIM25 (S2 Fig). Clones D and F are designated TRIM25^lo^. Clone E is wild type and has similar TRIM25 protein expression as the parental *ZC3HAV1*-knockout 293T cells (S2 Fig). Both TRIM25^lo^ clones (D and F) were tested in the subsequent experiments and showed similar results. We transfected TRIM25^lo^ cells with ZAP-expressing constructs and infected them with SINV Toto1101/Luc. TRIM25 knockdown significantly enhanced SINV Toto1101/Luc replication in the presence of ZAPS and ZAPL overexpression (Fig 5A). Furthermore, we co-transfected TRIM25^lo^ cells with TRIM25- and ZAP-expressing constructs to determine the antiviral effect of the different TRIM25 and ZAP combinations. FL TRIM25 alone inhibited SINV replication, likely due to overexpression (Fig 5B). We found that FL TRIM25 enhanced the activity of ZAPS more than the RING- and CCD-deficient TRIM25 mutants at both high MOI (Fig 5B) and low MOI (S3A Fig), in the context of similar expression levels of FL and mutant TRIM25 proteins (S3B Fig). This data suggests that both the E3 ligase activity and oligomerization of TRIM25 are important for the activation of ZAP. However, FL TRIM25 does not significantly enhance SINV inhibition by ZAPL (Fig 5B and S3A Fig), which hints at potential isoform-specific differences in the ZAP-TRIM25 synergy.

### TRIM25 mediates ubiquitination of ZAPS and ZAPL

**Fig 6:**
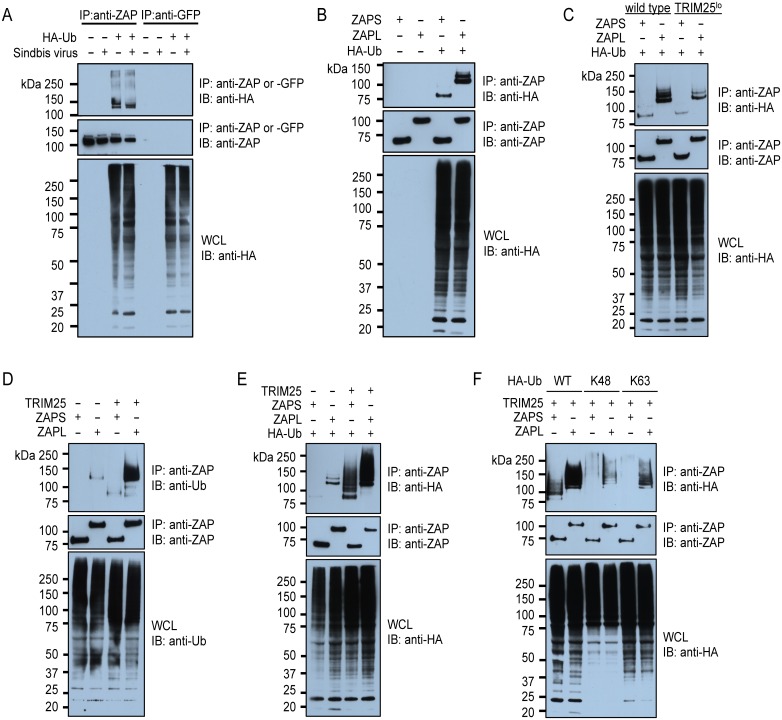
Both ZAPS and ZAPL are ubiquitinated by TRIM25. Cells were lysed in denaturing conditions to ensure pulldown of ZAP only and not ZAP-associated proteins. **(A)** WCL of 293T cells transfected with vector expressing HA-tagged ubiquitin (Ub), and mock infected or infected with the SINV Toto1101 strain (MOI = 1) for 18 hours were used for immunoprecipitation of endogenous ZAP with an anti-ZAP antibody and immunoblotting. The level of HA-tagged Ub in the ZAP pulldown is shown. The data is representative of 3 independent experiments. **(B)** WCL of *ZC3HAV1*-knockout 293T cells transfected with vectors expressing HA-tagged Ub, and ZAPS or ZAPL were used for immunoprecipitation of overexpressed ZAP with an anti-ZAP antibody and immunoblotting. The level of HA-tagged Ub in the ZAP pulldown is shown. The data is representative of 3 independent experiments. **(C)** WCL of wild type and TRIM25^lo^ *ZC3HAV1*-knockout 293T cells transfected with vectors expressing HA-tagged Ub, and ZAPS or ZAPL were used for immunoprecipitation with an anti-ZAP antibody and immunoblotting. The data is representative of 2 independent experiments performed on both clones D and F. Only data for clone D is shown here. **(D)** WCL of *ZC3HAV1*-knockout 293T cells transfected with vectors expressing ZAPS or ZAPL, and/or V5-tagged TRIM25 were used for immunoprecipitation with an anti-ZAP antibody and immunoblotting. The level of endogenous Ub in the ZAP pulldown is shown. The data is representative of 3 independent experiments. **(E)** WCL of *ZC3HAV1*-knockout 293T cells transfected with vector expressing HA-tagged Ub, ZAPS or ZAPL, and/or V5-tagged TRIM25 were used for immunoprecipitation with an anti-ZAP antibody and immunoblotting. The level of HA-tagged Ub in the ZAP pulldown is shown. The data is representative of 2 independent experiments. **(F)** WCL of *ZC3HAV1*-knockout 293T cells transfected with vector expressing HA-tagged wild type (WT), K48 or K63 Ub, ZAPS or ZAPL, and/or V5-tagged TRIM25 were used for immunoprecipitation with an anti-ZAP antibody and immunoblotting. The level of HA-tagged WT or mutant Ub in the ZAP pulldown is shown. The data is representative of 2 independent experiments.

Since the E3 ligase-defective TRIM25 (ΔRING) failed to stimulate the activity of ZAPS (Fig 5B), we hypothesized that TRIM25 might act by ubiquitinating ZAP and/or other host proteins. First, we asked whether ZAP is ubiquitinated. 293T cells transfected with a construct expressing HA-tagged ubiquitin were lysed under denaturing conditions to disrupt protein-protein interactions, and endogenous ZAP was immunoprecipitated with an anti-ZAP polyclonal antibody and probed with an anti-HA antibody. A control anti-GFP antibody of the same species as the anti-ZAP antibody was used to check for non-specific pulldown. We found that ZAP was modified by ubiquitination at baseline and upon SINV infection (Fig 6A). When ZAPS and ZAPL were overexpressed in the *ZC3HAV1*-knockout 293T cells in the presence of HA-tagged ubiquitin and subject to immunoprecipitation, both ZAP isoforms were found to be modified by ubiquitin, suggesting that the modified lysine(s) are likely shared by both isoforms and located in ZAPS (Fig 6B). However, ZAPL is more polyubiquitinated than ZAPS, which hints to additional ubiquitination sites in the PARP-like domain. In addition, we asked whether TRIM25 is implicated in the ubiquitination of ZAP and determined the effect of both endogenous and overexpressed TRIM25 on ZAP ubiquitination level. Both ZAPS and ZAPL ubiquitination in the TRIM25^lo^ cells was reduced compared to that in the parental TRIM25 sufficient *ZC3HAV1*-knockout cells (Fig 6C). Consistent with that, overexpressed TRIM25 dramatically increased the level of modification of ZAP isoforms by both endogenous (Fig 6D) and overexpressed ubiquitin (Fig 6E). Furthermore, we determined which linkage type of polyubiquitin TRIM25 induces on ZAP by overexpressing ubiquitin mutants, in which all the lysine residues are mutated except for K48 or K63. We immunoprecipitated ZAP isoforms that were co-expressed with TRIM25, and HA-tagged wild type or mutant (K48, K63) ubiquitin, and found that both ZAPS and ZAPL were ubiquitinated by both K48- and K63-linked polyubiquitin (Fig 6F). Together, these data suggest that TRIM25 is responsible for ZAP modification.

Next, the lysine residues in ZAPS that were predicted to be ubiquitinated with medium to high confidence by UbPred and CKSAAP_UbSite were changed to arginine residues individually (K226R, K296R, K314R, K401R, K416R, K448R, and K629R) or in combination (K296R/K448R, 7UbΔ) to determine whether potential ubiquitination at any of these sites affects ZAP’s antiviral activity. Moreover, a previous study, in which a global approach was taken to identify all the ubiquitinated proteins in the cell, reported a tryptic peptide with a ubiquitinated lysine in ZAPL (EEGK_783_(glygly)LLFYATSR) [32]. We confirmed that this residue is ubiquitinated using a targeted mass spectrometry approach and the lysine was mutated to arginine in ZAPL (K783R). When we co-expressed the panel of ZAP ubiquitination site mutants with TRIM25 and immunoprecipitated ZAP to determine the level of modification, we found that the mutations diminished the level of ZAP ubiquitination to various degrees (S4A Fig). Most importantly, introduction of all 7 mutations at the same time almost completely abrogated ZAP ubiquitination (refer to “S 7UbΔ” in S4A Fig). However, the ZAPS 7UbΔ mutant was still capable of inhibiting SINV replication (S4B Fig). Our data suggests that TRIM25 upregulates ZAP’s antiviral function not by modifying ZAP, but potentially other host factors.

It is possible that ZAP changes the interactome of TRIM25, which ubiquitinates and functionally modulates these interacting partners resulting in an antiviral state. To test this hypothesis, we co-immunoprecipitated TRIM25 in the absence or presence of ZAPS, of which the antiviral activity was more affected by the E3 ligase function of TRIM25 (Fig 5B), and identified by mass spectrometry proteins that interacted significantly more or less with TRIM25 upon ZAPS expression (S5 Fig). We found that most of these ZAP-mediated TRIM25 interacting partners are involved in mRNA metabolism and translation (S3 Table), which are cellular processes known to be affected by ZAP. However, the change in abundance of most of these proteins is not dramatic (S5 Fig), suggesting that the ZAP-mediated effects on TRIM25 targets might lie in their ubiquitination status. Taken together, we have shown that TRIM25 ubiquitinates ZAP, and that ZAP changes the interactions of TRIM25 with other host proteins that might contribute to the antiviral effects of the ZAP-TRIM25 synergy. Further work is required to determine whether these TRIM25 interacting partners are ubiquitinated and the functional consequences of their modification by TRIM25.

### TRIM25 plays a critical role in ZAP inhibition of viral translation

**Fig 7:**
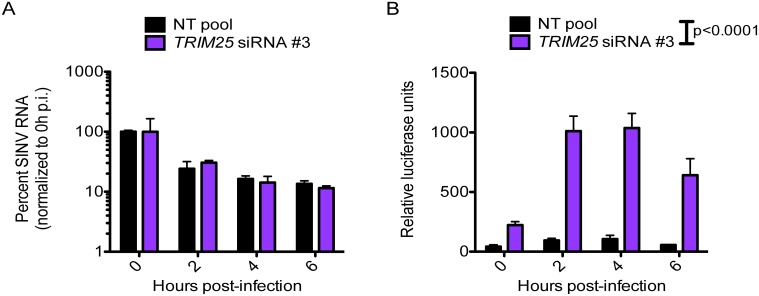
ZAP synergizes with TRIM25 to block SINV translation. T-REx-hZAP cells were transfected with *TRIM25*-targeting or NT pool siRNA, induced for ZAPS expression, and infected with a temperature-sensitive SINV that expresses luciferase and is unable to replicate at 40°C (Toto1101/Luc:ts6; MOI = 10). Following 1 hour of adsorption with virus at 37°C, cells were moved to 40°C, washed, and lysed at 0, 2, 4 and 6 h p.i. for measurement of **(A)** viral RNA by RT-qPCR, and **(B)** luciferase activity, which represents translation of the incoming viral genome. The data is representative of 2 independent experiments.

In order to further elucidate the mechanism by which TRIM25 synergizes with ZAP, we investigated the effects of TRIM25 on different ZAP activities that were previously reported (reviewed in [15]). We knocked down TRIM25 in T-REx-hZAP cells and infected them with a temperature sensitive SINV that is unable to replicate at the non-permissive temperature. Since the antiviral mechanisms of ZAP include targeting of viral RNAs for degradation by the exosome complex and translational inhibition, we measured the level of SINV RNA and translation of the incoming viral genome over the course of infection. We found that the kinetics of viral RNA degradation was similar in the presence or absence of TRIM25 (Fig 7A). However, ZAP’s ability to block SINV translation was significantly reduced by about 1 log in the absence of TRIM25 (Fig 7B; two-way ANOVA: p<0.0001), mirroring the magnitude of SINV inhibition upon TRIM25 knockdown in the same cells (Fig 3A). This data suggests that the mechanism by which TRIM25 enhances ZAP activity is through viral translational inhibition.

## Discussion

We reported in this study that ZAP requires multiple host factors for its maximal antiviral activity. We identified novel partners of ZAP that are normally important for a range of cellular processes, such as membrane ion permeability, innate immune signaling, and post-translational protein modification. Among the hits, JAK1 is a kinase important for signaling of the type I IFN receptor and can potentially act by augmenting the stimulatory effects of ZAP on the RIG-I pathway. On the other hand, KCNH5 is an outward rectifying potassium channel and it is not clear how it can stimulate ZAP’s function. It has been shown that reduction of the intracellular K+ concentration can activate the NLRP3 inflammasome, linking ion efflux to innate immunity [33]. It is interesting to note that known interacting partners of ZAP, such as RIG-I or components of the exosome complex, were not hits in the screen, although that is likely largely dependent on the type of assay used and the basal expression levels of genes that are knocked down. Moreover, although MAP3K14 was previously found to be synergistic with ZAP in an ISG overexpression screen [20], it was not a hit in our confirmatory screen. It is likely that synergistic effects are less pronounced in our screen where endogenous levels of proteins are being interrogated and the effect from silencing of one gene might be compensated by a homologous gene or another ZAP partner.

Our study shows for the first time that both ZAP isoforms are ubiquitinated, adding to the existing body of work on post-translational modifications of ZAP. Previous studies have shown that in addition to being post-translationally modified itself, ZAP is implicated in the modifications of other cellular and viral proteins. In some of these cases the modification regulates the function of ZAP or other proteins. For example, the C-terminal end of ZAPL is prenylated, and mutation of this prenylation site reduces the anti-SINV activity of ZAPL to a level similar to that of ZAPS, which is normally not prenylated [12]. It is possible that prenylation positions ZAPL in membrane compartments, allowing it to target viruses with an endocytic life cycle step. In addition, phosphorylation of ZAP by glycogen synthase kinase 3β positively modulates ZAP activity potentially by enhancing its ability to inhibit mRNA translation [34]. Moreover, when cells are stressed, ZAP localizes to cytoplasmic stress granules and is modified by poly-ADP-ribosylation [35]. Modified ZAP is implicated in the poly-ADP-ribosylation of Ago2, which correlates with derepression of miRNA-mediated translational silencing of cellular transcripts [13, 35]. In particular, derepression of ISG transcripts results in viral inhibition, as demonstrated for HSV-1 and influenza A virus [13]. ZAP, specifically the long isoform, is also implicated in the poly-ADP-ribosylation and ubiquitination of influenza viral PB2 and PA polymerase proteins and their subsequent degradation [36]. Interestingly, another study reports that ZAPS inhibits influenza protein expression and is antagonized by the viral NS1 protein [37]. A NS1 mutant that lacks the ability to suppress the E3 ligase activity of TRIM25 [38] also loses the ability to antagonize ZAPS [37], suggesting that ZAPS could inhibit influenza virus through a mechanism that requires TRIM25-mediated ubiquitination, which is antagonized by NS1.

Although we clearly demonstrate here that TRIM25 is implicated in ZAP modification (Fig 6C–6F), mutagenesis of ZAP ubiquitination sites does not impact its antiviral function (S4 Fig). One plausible explanation is that the E3 ligase activity of TRIM25 enhances ZAP function (Fig 5B) by ubiquitinating other host factors. It is likely that ZAP affects the interactions of TRIM25 with other proteins, resulting in changes of their modification and function and hence remodeling of the antiviral proteome in the cell. Since ZAP and TRIM25 synergize to block viral translation (Fig 7B), it is intriguing yet not unexpected to find that most of the TRIM25 interacting partners affected by ZAP are involved in mRNA metabolism and translation (S3 Table). Future work determining the ubiquitination status of these proteins will shed light on the impact of TRIM25 E3 ligase activity on the antiviral effects of ZAP.

Alternatively, TRIM25 and ZAPS can synergize to positively modulate the activity of RIG-I, leading to heightened innate immune signaling. About half of the TRIM family members have been shown to enhance innate immune responses and both ZAPS and TRIM25 physically interact with RIG-I to stimulate type I IFN response [14, 23, 39]. However, whether there is a requirement for RIG-I in ZAP function and vice versa are not settled. Inhibition of the RIG-I pathway and knockdown of RIG-I fail to abrogate ZAP-mediated HBV and XMRV inhibition, respectively [5, 7]. Furthermore, RIG-I-dependent production of type I IFN and cytokines are not reduced in ZAP-deficient primary mouse cells [40]. Hence, the contribution of TRIM25 and ZAP synergy to innate immunity warrants further investigation.

Our data also suggest differences in the interaction between TRIM25 and ZAPS and ZAPL, consistent with previous studies demonstrating distinct activities for the ZAP isoforms. TRIM25 was dramatically modified in the presence of ZAPS, although these modified forms were not associated with ZAPS (Fig 4B). On the other hand, ZAPL expression did not result in extensive modification of TRIM25 and more TRIM25 was found to associate with ZAPL (Fig 4B). This suggests that in addition to the shared domains in ZAPS and ZAPL, ZAPL might interact with TRIM25 through its PARP-like domain. These observations argue that ZAP interacts primarily with unmodified TRIM25, although the expression of the ZAP isoforms differentially regulates TRIM25 post-translational modification. Based on these observations, we postulate that ZAP might affect the ubiquitination status of TRIM25 and as a result modulate the innate immune response. It has been shown that ubiquitin-specific peptidase 15 deubiquitinates TRIM25, leading to its stabilization and sustained RIG-I signaling [41]. However, when we performed mass spectrometry on the immunoprecipitates of TRIM25, we did not see a significant increase in ubiquitinated TRIM25 peptides in the presence of overexpressed ZAPS. It is possible that the increased modification of TRIM25 is due to something other than ubiquitin. Future studies are needed to determine the impact of ZAP isoforms on post-translational modification of TRIM25. In addition, even though endogenous TRIM25 is required for the function of both ZAPS and ZAPL (Fig 5A), overexpressed TRIM25 stimulates the antiviral activity of ZAPS to a greater extent than that of ZAPL (Fig 5B), which is likely due to ZAPL’s greater baseline inhibitory effect compared to ZAPS in the absence of TRIM25.

In conclusion, our study has uncovered a novel requirement for TRIM25 in ZAP function and elucidated the mechanism of this synergy. Recent outbreaks, such as chikungunya virus (Alphavirus) in the Caribbean and U.S., Ebola virus (Filovirus) in West Africa, and Zika virus (Flavivirus) in South and Central America, prompts the development of therapeutic strategies for disruption of crucial virus-host interactions. Given that the replication of these viruses greatly depend on the host factor repertoire of the target cells, our study is highly relevant and advances our understanding of host factor contributions to innate immune responses.

## Materials and Methods

### Cells, plasmids, viruses, and infections

T-REx-rZAPC88R cells with tetracycline-inducible protein expression of the rat ZAP C88R mutant were previously described [18, 28]. To generate the T-REx-hZAP cell line, the short isoform of human *ZC3HAV1* was amplified from the ATCC clone 7521231 (deposited by The I.M.A.G.E. Consortium) and restriction sites HindIII and SacII were added using primers (5’-GGGAAGCTTGCCACCATGGCGGACCCGGAGGTGTGCTGCTTC-3’ and 5’-GCGGATCCGCGGCTCTGGCCCTCTCTTCATCTGCTGCAC-3’). The HindIII/SacII digested PCR product was then cloned into pcDNA4/TO/myc-hisB to generate pcDNA4/TO/hZAP-myc-hisB. We stably transfected T-REx-293 cells (Thermo Fisher Scientific) with pcDNA4/TO/hZAP-myc-hisB and selected for Zeocin resistance. A single clone with good induction of hZAP expression (6C5) was selected and expanded. T-REx-rZAPC88R and T-REx-hZAP were cultured in Dulbecco's Modified Eagle Medium‎ (DMEM) supplemented with 10% fetal bovine serum (FBS), 5 μg/ml blasticidin, and 200 μg/ml Zeocin. *ZC3HAV1*-knockout 293T (clone 89) was obtained from Dr. Akinori Takaoka at Hokkaido University [14]. Wild type and TRIM25^lo^ *ZC3HAV1*-knockout clones (see below for details), *ZC3HAV1*-knockout clone, and 293T cells were cultured in DMEM supplemented with 10% FBS.

Total RNA was isolated from 293T cells by RNeasy Mini Kit (Qiagen), and reverse transcribed using SuperScript III First-Strand Synthesis System for RT-PCR (Invitrogen) and specific primers targeting the 3’UTR regions of the short and long isoforms of human ZAP (ZAPS 3’UTR: 5’-ACTTGATGAGCCCAGGGCATG-3’; ZAPL 3’UTR: 5’-GTCTGCGGCAATTTAGTTCTG-3’). ZAPS and ZAPL were amplified from 293T cDNA using primers (ZAPS: 5’-GTTTTGTACAGCCACCATGGCGGACCCGGAGGTG-3’ and 5’-GGTAGCGGCCGCTTACTCTGGCCCTCTCTTCATC-3’; ZAPL: 5’-GTTTTGTACAGCCACCATGGCGGACCCGGAGGTG-3’ and 5’-GGTA GCGGCCGCCTAACTAATCACGCAGGCTTTG-3’) and cloned into the BsrGI and NotI sites of a modified pTRIPZ construct (Open Biosystems) under the control of the CMV promoter. The 3’ ends of ZAPS and ZAPL were swapped into SmaI and XhoI sites of pTRIP-RFP-NZAP [42] to generate pTRIP constructs expressing N-terminally RFP-fused ZAPS and ZAPL. V5-tagged FL TRIM25 and derivatives (domains alone: RING, B box/CCD and SPRY; domain deletion mutants: ΔRING and ΔCCD) were expressed from a modified pIRES-puro vector encoding a C-terminal V5 tag as previously described and were gifts from Jae U. Jung [23, 38]. pcDNA3.1(HA-Ub)_6_ was previously described [43]. pRK5-HA-Ubiquitin-K48 (all the lysines in ubiquitin are mutated except K48), pRK5-HA-Ubiquitin-K63 (all the lysines in ubiquitin are mutated except K63) and pRK5-HA-Ubiquitin-WT were gifts from Ted Dawson (Addgene plasmid #17605, 17606 and 17608) [44]. pSpCas9(BB)-2A-Puro (PX459) was a gift from Feng Zhang (Addgene plasmid # 48139) [31].

SINV (Toto1101), and SINV expressing firefly luciferase (Toto1101/Luc and Toto1101/Luc:ts6) or EGFP (TE/5’2J/GFP) have been previously described [3, 45, 46]. Stocks were generated in baby hamster kidney 21 (BHK-21; ATCC) cells as previously described [3]. The Toto1101/Luc stock used for the screen was concentrated using polyethylene glycol with an average molecular weight of 6,000 (Fluka) as described [47]. Viral titers for multiplicity of infection (MOI) calculations were determined in BHK-21 cells. Viral infections were performed as previously described [3].

### Genome-wide siRNA screen

For the primary ZAP cofactor screen, siRNAs from the siGENOME library targeting the whole human genome (Dharmacon; pools of 4 siRNAs per gene) were pre-arrayed in fifty-eight 384-well plates, mixed with DharmaFECT 1 Transfection Reagent (diluted 1:100), and transferred to 384-well assay plates (5 μl/well; 25 nM final) with a Janus automated workstation (PerkinElmer). The entire screen was carried out in triplicate by reverse transfection. siGENOME NT and *ZC3HAV1*-targeting siRNA smartpools were used as negative and positive controls (5 wells per control per plate; see the position of the controls in S1 Table). A total of 7500 T-REx-hZAP cells were seeded in DMEM with 3% FBS, 5 μg/ml blasticidin, and 200 μg/ml Zeocin (25 μl/well) with a MultiDrop Combi liquid dispenser (Thermo Fisher Scientific). One day post-plating, ZAP expression was induced by addition of 1 μg/ml doxycycline (5 μl/well). Since removing the media from 384-well plates and adding virus inoculum in a minimal volume to allow adsorption was not feasible, cells were infected with a highly concentrated PEG-precipitated SINV stock at a MOI of ~200 (5 μl/well) two days following siRNA treatment. The MOI was calculated based on titration of the viral stock under standard infection conditions, and due to dilution was likely much lower under the conditions used in the screen. Twenty-four hours p.i., cells were lysed with Steady-Glo Luciferase Assay Substrate (Promega; 20 μl/well) and luminescence measured on an EnVision plate reader (PerkinElmer). For each well, the raw luciferase value reflects the level of SINV infection. The raw luciferase values and the plate median from each of the 384-well plates were used to calculate the median absolute deviation (MAD): 1.4826 x MEDIAN (each raw value − plate median). The robust Z score was then calculated by dividing the difference between each raw luciferase value and the plate median by MAD [26]. Normalized percent activation (NPA) was also calculated for each well as follows: 100*(X_i_−μ_c-_)/(μ_c+_−μ_c-_), where X_i_ = raw luciferase value, μ_c+_ = mean positive controls and μ_c-_ = mean negative controls. The entire screen was performed in triplicate and the Z' and strictly standardized mean difference (SSMD) were calculated for every plate [26]. A Z' factor of >0.5 and a SSMD of >3 were used as cutoffs for assay validation [26, 48, 49]. Those wells with the highest NPA and robust Z score >3 in all three replicates were considered ‘hits’.

For the secondary screen, Silencer siRNAs (Ambion; 3 individual siRNAs per gene) were tested in 384-well plates in triplicate at two different concentrations: 6.25 nM (the concentration of each single siRNAs in the original Dharmacon pools) and 25 nM (the concentration of the original Dharmacon pools used in the primary screen). In order to rule out ZAP-independent effects of the top hits, the secondary screen was carried out in two different cells lines: T-REx-hZAP and T-REx-rZAPC88R. Genes with an average Z score of >3 for at least two out of the three siRNA sequences were considered ‘hits’.

### Bioinformatics and statistical analyses

Pathway analysis was performed using Enrichr [50]. All genes with an average robust Z score >3 were normalized using -5 as the minimum (essentially the value of the negative controls) and the mean value for *ZC3HAV1* as the maximum, scaled between 0 and 1, and exported as a comma-separated table with the normalized score and gene symbol, a format recognized by Enrichr. Genes belonging to pathways that were significantly enriched and overlapping with the list of genes with the highest NPA and robust Z score >3 in at least 1 out of 3 replicates were validated in the secondary screen. Haystack analysis was performed to identify the most statistically significant genes whose predicted knockdown via off-target effects was correlated with the phenotypes observed in the primary screen [27]. Differences between experimental conditions during the course of infection were determined using two-way ANOVA. Differences between experimental conditions at a single time point were determined using an unpaired, two-tailed Student’s t-test with a 95% confidence interval.

### Validation of candidate ZAP cofactors

For our validation studies, mammalian cells (T-REx-hZAP, 293T and *ZC3HAV1*-knockout 293T cells) were reverse transfected with 25 nM Silencer siRNAs targeting *TRIM25*, *KCNH5*, *JAK1*, *ZC3HAV1* (Ambion) that were used in the secondary screen or a C911 control for *TRIM25* (Ambion) using DharmaFECT 1 Transfection Reagent (diluted 1:100). siGENOME NT and *ZC3HAV1*-targeting siRNA smartpools (Dharmacon) were used as negative and positive controls. Sequences for TRIM25-targeting Ambion Silencer and the corresponding C911 control siRNAs were as follows: *TRIM25* siRNA #3 (5’-CCCUGAGGCACAAACUAACtt-3’ and 5’-GUUAGUUUGUGCCUCAGGGtg-3’); and *TRIM25* #3-C911 (5’-CCCUGAGGGUGAAACUAACtt-3’ and 5’-GUUAGUUUCACCCUCAGGGtg-3’). One day post-transfection, ZAP expression was induced in T-REx-hZAP cells by 1 μg/ml doxycycline. Two days post-transfection, the media was removed and T-REx293-hZAP, 293T and *ZC3HAV1*-knockout 293T cells were infected with SINV Toto1101/Luc at a MOI of 0.01 or 10 in a minimal inoculum volume. 24 hours p.i., cells were lysed with 1x Cell Culture Lysis Reagent (Promega) and luminescence measured on a Synergy Neo plate reader. For time course experiments, cells were lysed at 6, 12, 24, and 40 hours p.i. and luciferase activity measured.

### Plasmid transfection

Cells were transfected in different combinations with constructs expressing V5-tagged TRIM25 (FL) or derivatives (RING, B box/CCD, SPRY, ΔRING, and ΔCCD), ZAPS or ZAPL, and HA-tagged wild type ubiquitin or mutants with X-tremeGENE 9 DNA Transfection Reagent (Roche Life Science) at a ratio of 3 μl reagent to 1 μg DNA. Total plasmid amount in co-transfections was kept constant by transfecting cells with empty vectors (pIRES-puro, pTRIPZ and pcDNA3.1).

### *TRIM25* targeting by CRISPR

Guide RNAs (gRNAs) targeting exon 1 of the human *TRIM25* gene were designed by the MIT Optimized CRISPR Design portal (http://crispr.mit.edu/), and two with the least predicted off-target effects (gRNA #1: GTCGCGCCTGGTAGACGGCG; gRNA #3: GAGCCGGTCACCACTCCGTG) were selected for cloning into the *Cas9*-expressing PX459 vector. Oligos containing the gRNA sequences (gRNA #1: 5’-CACCGGTCGCGCCTGGTAGACGGCG-3’ and 5’-AAACCGCCGTCTACCAGGCGCGACC-3’; gRNA #3: 5’-CACCGGAGCCGGTCACCACTCCGTG-3’ and 5’-AAACCACGGAGTGGTGACCGGCTCC-3’) were ligated and cloned into PX459 linearized with BbsI. *ZC3HAV1*-knockout 293T cells were transiently transfected with PX459 expressing gRNA #1 or 3, and one day after transfection selected under 1 μg/ml puromycin for 2 days to eliminate cells that were not transfected. Surviving cells were then counted, diluted to 0.5 cell/well in a 96-well plate and seeded in 10% FBS DMEM. Single cell clones were marked and allowed to expand. Several clones per gRNA #1 were treated with or without puromycin and the ones that were sensitive to puromycin, suggesting that the clones had not integrated the gRNA-expressing vector, were harvested for immunoblot analysis to evaluate TRIM25 expression. Two knockdown clones (D and F) and one wild type clone (E) were selected (see S2 Fig). Genomic DNA was isolated from these clones, and a 600bp sequence flanking the gRNA targeting site was amplified by PCR and cloned into TOPO vector (Thermo Fisher Scientific). Sequencing of TOPO clones confirmed that all three chromosomes were targeted in clones D and F resulting in insertions and/or deletions in exon 1 of *TRIM25*, while clone E is wild type.

### Immunoprecipitation (IP) assay

Transfected or untransfected cells in 6-well plates were collected and then lysed in 0.5% NP40 buffer (for co-IP; 10 mM HEPES, pH 7.5, 150 mM KCl, 3 mM MgCl_2_, 0.5% NP-40) or 0.5% SDS buffer (for denaturing ZAP IP; 0.5% SDS, 50 mM Tris-HCl, pH 7.5, 200 mM NaCl, 1 mM EDTA) supplemented with a complete protease inhibitor cocktail (Roche) and 0.1 mM PMSF (Sigma). For co-IP of endogenous TRIM25 and ZAP, 300 μl of WCL were incubated with 1 μg of anti-TRIM25 antibody overnight at 4 °C, and then with 40 μl Protein A Dynabeads (Invitrogen) for 2 h at 4°C. For co-IP of overexpressed TRIM25 and ZAP, 5.25 μg of anti-NZAP antibody was covalently crosslinked to 70 ul Protein A Dynabeads by BS^3^ (Thermo Fisher Scientific) and incubated with 300 μl of cell lysate at 4 °C for 4h. Immunoprecipitates were washed 3 times with 0.5% NP40 buffer, followed by two washes with 0.05% NP40 buffer. For denaturing IP, 300 μl WCL were diluted into 1X TNA buffer (0.25% Triton, 50 mM Tris-HCl, pH 7.5, 200 mM NaCl, 1 mM EDTA) + 2 mg/ml BSA, incubated with 1 μg anti-ZAP, or anti-GFP antibody overnight at 4°C, and then with 40 μl Protein A Dynabeads (Invitrogen) for 2 h at 4°C. Immunoprecipitates were washed 3 times with 1X TNA buffer + 2 mg/ml BSA. Bound proteins were eluted with SDS loading buffer and boiled for 5 min.

### Immunoblot analysis

Polypeptides were resolved by SDS–polyacrylamide gel electrophoresis (SDS–PAGE) and transferred to a nitrocellulose membrane (GE Healthcare). Immunodetection was achieved with 1:5000 anti-ZAP (ab154680; Abcam), 1:5000 anti-NZAP (mouse monoclonal 23D1.1; see below), 1:5000 anti-V5 (MA5-15253; Thermo Fisher Scientific), 1:5000 anti-TRIM25 (610570; BD Biosciences), 1:1000 anti-HA (clone 3F10; Roche), 1:500 anti-ubiquitin (P4D1; Santa Cruz Biotechnology), or 1:50,000 anti-actin-HRP (A3854; Sigma) antibodies. The primary antibodies were detected with 1:20,000 goat anti-mouse HRP (115-035-146; Jackson ImmunoResearch), 1:20,000 goat anti-rabbit HRP (31462; Thermo Fisher Scientific), or 1:20,000 donkey anti-rat HRP (712-035-153; Jackson ImmunoResearch). Mouse monoclonal antibodies to rat NZAP previously generated [51] were screened for cross-reactivity to human NZAP. The clone 23D1.1 was submitted for production and purification by Cell Essentials. Anti-GFP antibody (rabbit polyclonal) was generated previously [52]. The proteins were visualized by ECL Prime Western Blotting Detection Reagent (GE Healthcare) or SuperSignal West Pico Chemiluminescent Substrate (Thermo Fisher Scientific).

### Quantitative reverse transcription PCR (RT-qPCR)

Total RNA was isolated from siRNA-treated cells using the RNeasy mini kit (Qiagen). 1 μg of input RNA was used as a template for reverse transcription using SuperScript III (Invitrogen, Carlsbad, CA) and random hexamers. RT-qPCR was performed using 5 μl of 10-fold-diluted cDNA and primers targeting JAK1 (5’-CCACTACCGGATGAGGTTCTA-3’ and 5’-GGGTCTCGAATAGGAGCCAG-3’), KCNH5 (5’-CCGTGTGGCTAGGAAACTGG-3’ and 5’-CAATGACCTCGTAGTCTCCGA-3’), and RPS11 (5’-GCCGAGACTATCTGCACTAC-3’ and 5’-ATGTCCAGCCTCAGAACTTC-3’ [53]) in a SYBR Green qPCR assay on the LightCycler 480 Real-Time PCR System (Roche Applied Sciences, Indianapolis, IN). qPCR conditions were as follows; initial denaturation step at 50°C for 2 min and 95°C for 10 min, then 45 cycles of 95°C for 15 sec, 56°C for 15 sec, and 72°C for 20 sec, and followed by a melting step of 95°C for 10s, 65°C for 10s and a 0.07°C/s decrease from 95°C, and a cooling step of 50°C for 5s. Transcript levels of JAK1 and KCNH5 were determined by normalizing the target transcript CT value to the CT value of the endogenous housekeeping RPS11 transcript. This normalized value was used to calculate the fold change relative to the average of cells treated with the NT siRNA control (CT method).

To determine SINV RNA levels in *TRIM25*-targeting or NT siRNA treated T-REx-hZAP cells over the course of infection with Toto1101/Luc:ts6, 1 μg of total cellular RNA was used in a one-step quantitative real-time PCR assay using primers and a Taqman probe targeting the nsP2 region of SINV. Primer pairs for SINV Taqman RT-qPCR are as follows: SINV nsP2 (forward): 5’-GGTAGCTCATTGGGACAACA-3’; SINV nsP2 (reverse): 5’-GCTGGAACACCGGAAATCTA-3’; SINV nsP2 Taqman probe (reverse): 5’-TGGCGTGATCGTACCCATACTTGC-3’. RNA was amplified using Lightcycler 480 RNA Master Hydrolysis Probes (Roche) under the following thermal conditions: RT at 63°C for 3 min; denaturation at 95°C for 30 s; 45 cycles of amplification at 95°C for 15 s, 60°C for 30 s, and 72°C for 1 s; and a final cooling step at 40°C for 10 s. Viral RNA copy number at each time point p.i. was then determined by comparing the threshold cycle (CT) value to a standard curve of serial 10-fold dilutions of purified *in vitro* transcribed SINV RNA. Viral RNA levels were normalized to that at 0 h p.i.

### Generation and functional assay of ZAP ubiquitination site mutants

ZAPS ubiquitination site mutants (K226R, K296R, K314R, K401R, K416R, K448R, K629R, and K296R/K448R) were generated by mutagenesis of pTRIP-RFP-ZAPS using the QuikChange II XL Site-Directed Mutagenesis Kit (Agilent Technologies). Primers for site-directed mutagenesis of ZAP were as follows: ZAPS K226R (5’-GCAAGCACATGCAGAGGAATCCCCCAGGGCC-3’ and 5’-GGCCCTGGGGGATTCCTCTGCATGTGCTTGC-3’); ZAPS K296R (5’-ACGATCTCACCCGCAGGTTCACGTATCTGGG-3’ and 5’-CCCAGATACGTGAACCTGCGGGTGAGATCGT-3’); ZAPS K314R (5’-CCTCAGGCTCGTCCAGGGCTACTGATCTTGG-3’ and 5’-CCAAGATCAGTAGCCCTGGACGAGCCTGAGG-3’); ZAPS K401R (5’-CTGTGACCACCAGAAGGGGCACAGGCTTGC-3’ and 5’-GCAAGCCTGTGCCCCTTCTGGTGGTCACAG-3’); ZAPS K416R (5’-GGATCATCAATGGCAGAAGTGGAACTCAGGACATCC-3’ and 5’-GGATGTCCTGAGTTCCACTTCTGCCATTGATGATCC-3’); ZAPS K448R (5’-CCAGATCCTTAAATTACAGAAGCACTAGCAGCGGTCACAG-3’ and 5’-CTGTGACCGCTGCTAGTGCTTCTGTAATTTAAGGATCTGG-3’); ZAP K629R (5’-GAGAAAGACAAACGGAGAAATTCAAACGTCGACTCTTC-3’ and 5’-GAAGAGTCGACGTTTGAATTTCTCCGTTTGTCTTTCTC-3’). ZAPS 7UbΔ was generated by cloning a 1.9 kb synthesized DNA fragment (IDT) containing all the lysine-to-arginine mutations into pTRIP-RFP-ZAPS. The ZAPL K783R mutant was generated by cloning an overlapping PCR fragment into the SalI and XhoI sites of ZAPL (PCR #1: 5’-GTTTGTCGACTCTTCATACCTGGAG-3’ and 5’-GGAGTCTTCCTTCTTCCTTCATCTG-3’; PCR #2: 5’-GAAGGAAGACTCCTATTTTATGCGAC-3’ and 5’-GGTACTCGAGCTAACTAATCACGCAGGCTTTG-3’). Clones were sequence verified and packaged into lentiviruses by co-transfection with Gag-Pol and VSV-G-expressing plasmids into 293T cells [20]. *ZC3HAV1*-knockout 293T were transduced with lentiviruses carrying RFP alone, ZAPS, ZAPL, and various ubiquitination site mutants, and 2 days later infected with TE/5’2J/GFP at a MOI of 10. Cells were harvested at 6–8 h p.i. and fixed in 1% paraformaldehyde for flow cytometry analysis. Data was acquired using a BD LSRII and FACS Diva software (version 8.0) and analyzed using FlowJo 8.8.7 (TreeStar Inc.). Percent infected (GFP+) cells in the transduced (RFP+ or RFP-tagged ZAP+) population was calculated for ZAPS, ZAPL and their mutants.

### Mass spectrometry (MS)

For the ZAP ubiquitination experiment, based on alignment of the ZAP IP Western Blot, SDS-PAGE gel bands corresponding to ZAP were excised and trypsinized as described previously [54]. For the co-immunoprecipitation experiment, TRIM25 and associated proteins were co-immunoprecipitated and eluted from anti-V5 antibody-crosslinked M-270 Epoxy Dynabeads (Thermo Fisher Scientific) using 8M Urea (GE Healthcare Life Sciences), reduced, alkylated and digested with Endopeptidase Lys-C (>4M Urea) for 6 hours followed by overnight trypsinization (>2M Urea).

Peptides were analyzed by reversed phase nano LC-MS/MS (Ultimate 3000 nano-HPLC system coupled to a Q-Exactive Plusor a Fusion Lumos mass spectrometer, operated in high/low mode, Thermo Fisher Scientific). Known (EEGK_783_(glygly)LLFYATSR [32]) and predicted ubiquitinated ZAP peptides were analyzed by parallel reaction monitoring (PRM) [55] while data dependent acquisition (DDA) was used for unknown ZAP ubiquitination sites. Peptides generated from the co-immunoprecipitation experiment were analyzed in DDA mode. All peptides were separated on a C18 column (12 cm / 75 μm, 3 μm beads, Nikkyo Tecnologies) at 300 nl/min with a gradient increasing from 2% Buffer B/98% buffer A to 40% buffer B/60% Buffer A in 37 min or 80 min (buffer A: 0.1% formic acid, buffer B: 0.1% formic acid in acetonitrile).

For data analysis, ubiquitination focused DDA data was extracted and queried against UniProts complete human proteome database (March 2016) concatenated with common contaminants [56] using Proteome Discoverer 1.4 (Thermo Fisher Scientific)/MASCOT 2.5.1 (Matrix Science). Protein N-terminal acetylation, oxidation of methionine, and di-glycine modification of lysine were allowed as variable modifications. In cases where proteins were reduced (DTT) and alkylated (iodoacetaminde), carbamidomethylation was included as a variable modification of cysteines and lysines. 10 ppm and 20 mDa were used as mass accuracy for precursors and fragment ions, respectively. Matched peptides were filtered using 1% False Discovery Rate calculated by Percolator [57] and in addition requiring that a peptide was matched as rank 1 and that precursor mass accuracy was better than 5 ppm. Data from the co-immunoprecipitation experiment, in biological triplicates, was analyzed by MaxQuant v.1.5.3.28 using match between runs. Search criteria similar to above were used. Fold differences were calculated based on label-free quantification (LFQ) values (http://www.ncbi.nlm.nih.gov/pubmed/24942700) while intensity-based absolute quantitation (iBAQ) values (https://www.ncbi.nlm.nih.gov/pubmed/21593866) were used to estimate abundance of different proteins. The statistical packet Perseus (https://www.ncbi.nlm.nih.gov/pubmed/27348712) was used for data analysis.

## Supporting Information

S1 Fig Validation of *TRIM25*, *ZC3HAV1*, *KCNH5* and *JAK1* silencing. Knockdown of **(A)** *TRIM25* and **(B)** *ZC3HAV1* by 3 individual siRNAs in T-REx-hZAP cells was validated by immunoblot analyses using the indicated antibodies. β-actin was used as a loading control. Pooled siRNAs that were NT or targeted *ZC3HAV1* were used as negative and positive controls for siRNA transfection, respectively. **(C)** Knockdown of *KCNH5* and *JAK1* by 3 individual siRNAs in T-REx-hZAP cells was validated by RT-qPCR. Mean fold change relative to the NT control pool treated cells ± standard deviation is plotted.
(TIF)

S2 Fig Validation of TRIM25 knockout in CRISPR clones. **(A)** CRISPR-targeting region in the genomic sequence of *TRIM25* is shown in clones D, E and F. Clone E has the wild type sequence. The alignment shown is in the same reading frame of the wild type TRIM25 protein. A red dash represents a deletion whereas a red nucleotide represents an insertion when compared to the wild type *TRIM25* sequence. **(B and C)** Protein level of TRIM25 in the parental *ZC3HAV1*-knockout 293T, and CRISPR clones E, D and F. Clone E has similar TRIM25 expression as the parental cells whereas TRIM25 expression is significantly lower in clones D and F (designated TRIM25^lo^) that are mutated for wild type TRIM25. β-actin was used as a loading control. Short and long exposures are shown in **(B)** and **(C)**, respectively.
(TIF)

S3 Fig Reconstitution of TRIM25^lo^ *ZC3HAV1*-knockout 293T cells with TRIM25 and mutants. TRIM25^lo^ *ZC3HAV1*-knockout 293T cells (clone D) were reconstituted with FL or truncated TRIM25 (ΔRING, ΔCCD) and/or ZAPS or ZAPL, and infected with Toto1101/Luc (MOI = 0.01) 2 days post-transfection. **(A)** Cell lysates in triplicate wells were harvested for measurement of luciferase activity at 24 h p.i. Relative luciferase units represent the level of SINV replication. Asterisks indicate statistically significant differences (Student’s t-test, *, p<0.05; **, p<0.005). **(B)** In a separate well, WCL were harvested for immunoblotting 2 days post-transfection. The levels of V5-tagged FL TRIM25 and mutants, ZAPS and ZAPL, and β-actin are shown.
(TIF)

S4 Fig Protein expression and activity of ZAP ubiquitination site mutants. **(A)** *ZC3HAV1*-knockout 293T cells were transfected with constructs expressing HA-tagged ubiquitin, V5-tagged TRIM25, and the panel of RFP-fused ZAP ubiquitination site mutants (lysine to arginine substitutions). WCL were harvested for immunoblotting 2 days post-transfection to check for the degree of ubiquitination of the ZAP mutants. **(B)** *ZC3HAV1*-knockout 293T cells were transduced with lentiviruses carrying the panel of RFP-fused ZAP ubiquitination site mutants (lysine to arginine substitutions) and 2 days later infected with TE/5’2J/GFP at a MOI of 10. Cells were harvested at 6–8 h p.i. and fixed for flow cytometry analysis. Percent infected (GFP+) cells in the transduced (RFP+) population for each mutant is shown here. The data is representative of 3 independent experiments. Asterisks indicate statistically significant differences between ZAPS and its mutants, or ZAPL and its mutants (Student’s t-test, *, p<0.05).
(TIF)

S5 Fig Volcano plot of TRIM25 interacting proteins upon ZAPS expression. V5-tagged TRIM25 and associated proteins were co-immunoprecipitated in the absence or presence of ZAPS, and identified and quantitated by LC-MS/MS. The x-axis indicates fold differences in samples with ZAPS compared to those without (Log_2_) while the y-axis indicates Student’s t-test calculated p-values (-Log_10_); each dot represents a unique protein identified by LC-MS/MS. Red filled circles mark proteins in the TRIM25 interactome that have more than one peptide identified and a linear-fold difference of ≥1.5 (Student’s t-test p< 0.05) in the presence of ZAPS. Fold differences are calculated from LFQ values, and the size of the filled circles indicates iBAQ values for the matched proteins where the largest circles suggest the most abundant proteins (see the section on MS in Materials and Methods).
(TIF)

S1 Table Results of the primary screen. The raw luciferase data collected from the T-REx-hZAP cell line, and the corresponding library gene list and controls are shown. Raw luciferase values are color-coded based on their magnitude (red: high; yellow: medium; green: low). Each column represents a different 384-well plate. A total of 58 plates were tested (H101-H158) in triplicates (A, B and C).
(XLSX)

S2 Table Results of the secondary screen. The raw luciferase data collected from T-REx-hZAP and T-REx-rZAPC88R cell lines and the corresponding library gene list are shown.
(XLSX)

S3 Table ZAP-mediated TRIM25 interactome. Host proteins that were significantly (Student’s t-test, p<0.05) up- or down regulated by 1.5 linear fold or more in TRIM25 pulldown in the presence of ZAPS are shown. The proteins with the most significant p-value are listed first. LFQ and iBAQ values were used to calculate fold differences of proteins between samples with and without ZAPS, and abundance of different proteins, respectively (see the section on MS in Materials and Methods).
(XLSX)
