# Supplementary figures and images for: TRIM25 Enhances the Antiviral Action of Zinc-Finger Antiviral Protein (ZAP)
Source: PLoS Pathog. 2017 Jan 6;13(1):e1006145. doi: 10.1371/journal.ppat.1006145 (PMC5245905; doi:10.1371/journal.ppat.1006145)

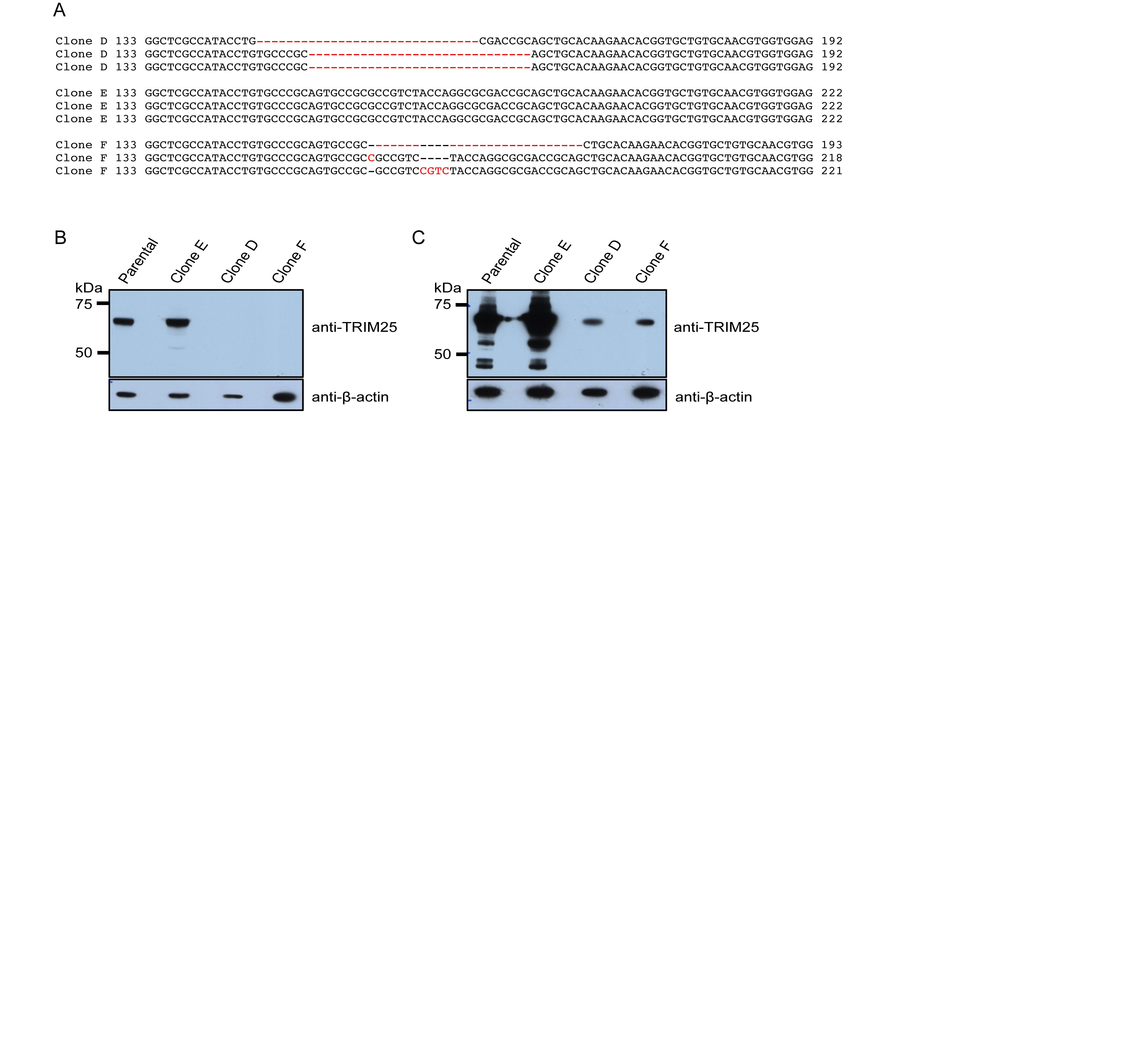

Supplement: S2 Fig — (A) CRISPR-targeting region in the genomic sequence of TRIM25 is shown in clones D, E and F. Clone E has the wild type sequence. The alignment shown is in the same reading frame of the wild type TRIM25 protein. A red dash represents a deletion whereas a red nucleotide represents an insertion when compared to the wild type TRIM25 sequence. (B and C) Protein level of TRIM25 in the parental ZC3HAV1-knockout 293T, and CRISPR clones E, D and F. Clone E has similar TRIM25 expression as the parental cells whereas TRIM25 expression is significantly lower in clones D and F (designated TRIM25lo) that are mutated for wild type TRIM25. β-actin was used as a loading control. Short and long exposures are shown in (B) and (C), respectively. (TIF) [file ppat.1006145.s002.tif]

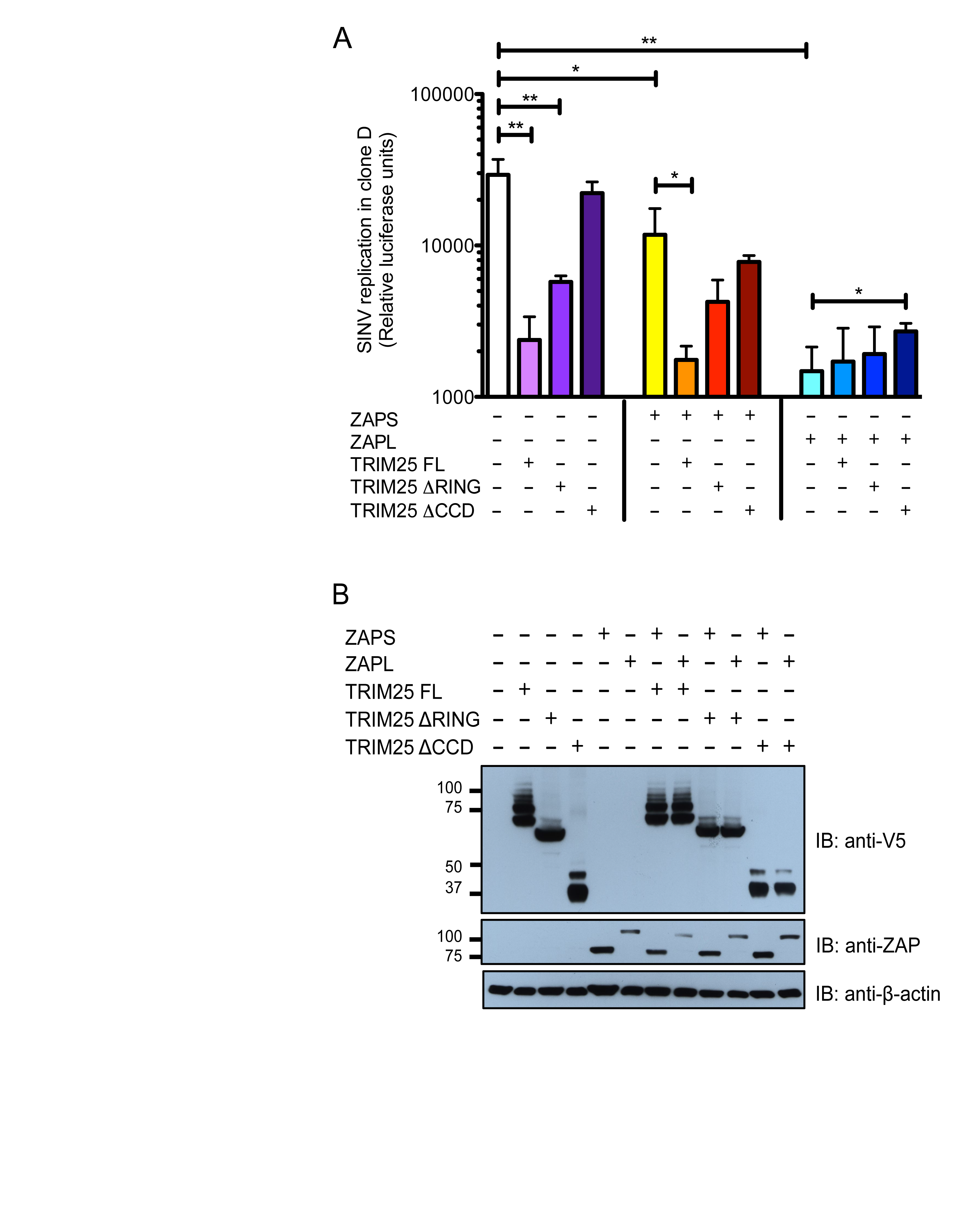

Supplement: S3 Fig — TRIM25lo ZC3HAV1-knockout 293T cells (clone D) were reconstituted with FL or truncated TRIM25 (ΔRING, ΔCCD) and/or ZAPS or ZAPL, and infected with Toto1101/Luc (MOI = 0.01) 2 days post-transfection. (A) Cell lysates in triplicate wells were harvested for measurement of luciferase activity at 24 h p.i. Relative luciferase units represent the level of SINV replication. Asterisks indicate statistically significant differences (Student’s t-test, *, p<0.05; **, p<0.005). (B) In a separate well, WCL were harvested for immunoblotting 2 days post-transfection. The levels of V5-tagged FL TRIM25 and mutants, ZAPS and ZAPL, and β-actin are shown. (TIF) [file ppat.1006145.s003.tif]

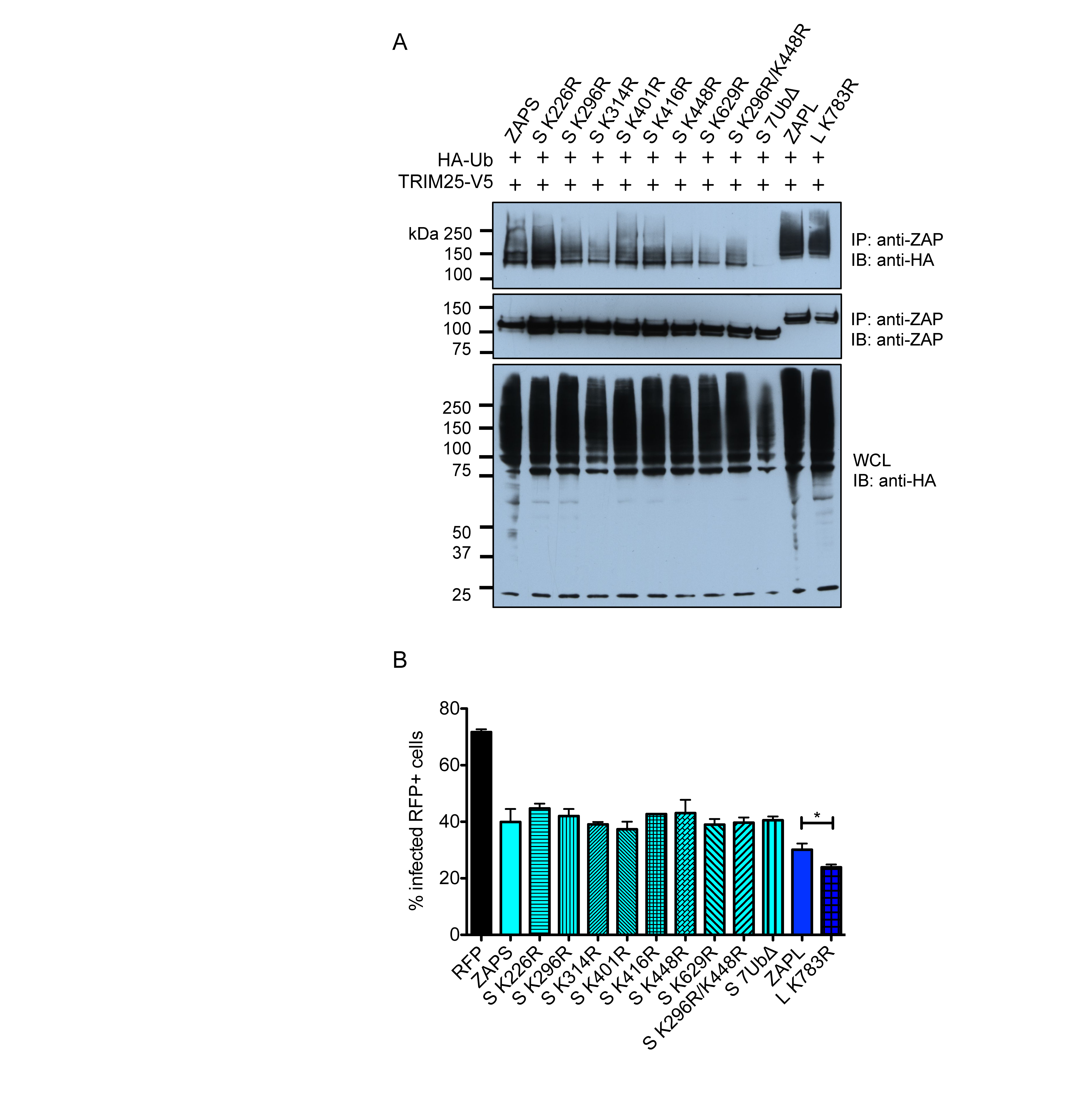

Supplement: S4 Fig — (A) ZC3HAV1-knockout 293T cells were transfected with constructs expressing HA-tagged ubiquitin, V5-tagged TRIM25, and the panel of RFP-fused ZAP ubiquitination site mutants (lysine to arginine substitutions). WCL were harvested for immunoblotting 2 days post-transfection to check for the degree of ubiquitination of the ZAP mutants. (B) ZC3HAV1-knockout 293T cells were transduced with lentiviruses carrying the panel of RFP-fused ZAP ubiquitination site mutants (lysine to arginine substitutions) and 2 days later infected with TE/5’2J/GFP at a MOI of 10. Cells were harvested at 6–8 h p.i. and fixed for flow cytometry analysis. Percent infected (GFP+) cells in the transduced (RFP+) population for each mutant is shown here. The data is representative of 3 independent experiments. Asterisks indicate statistically significant differences between ZAPS and its mutants, or ZAPL and its mutants (Student’s t-test, *, p<0.05). (TIF) [file ppat.1006145.s004.tif]

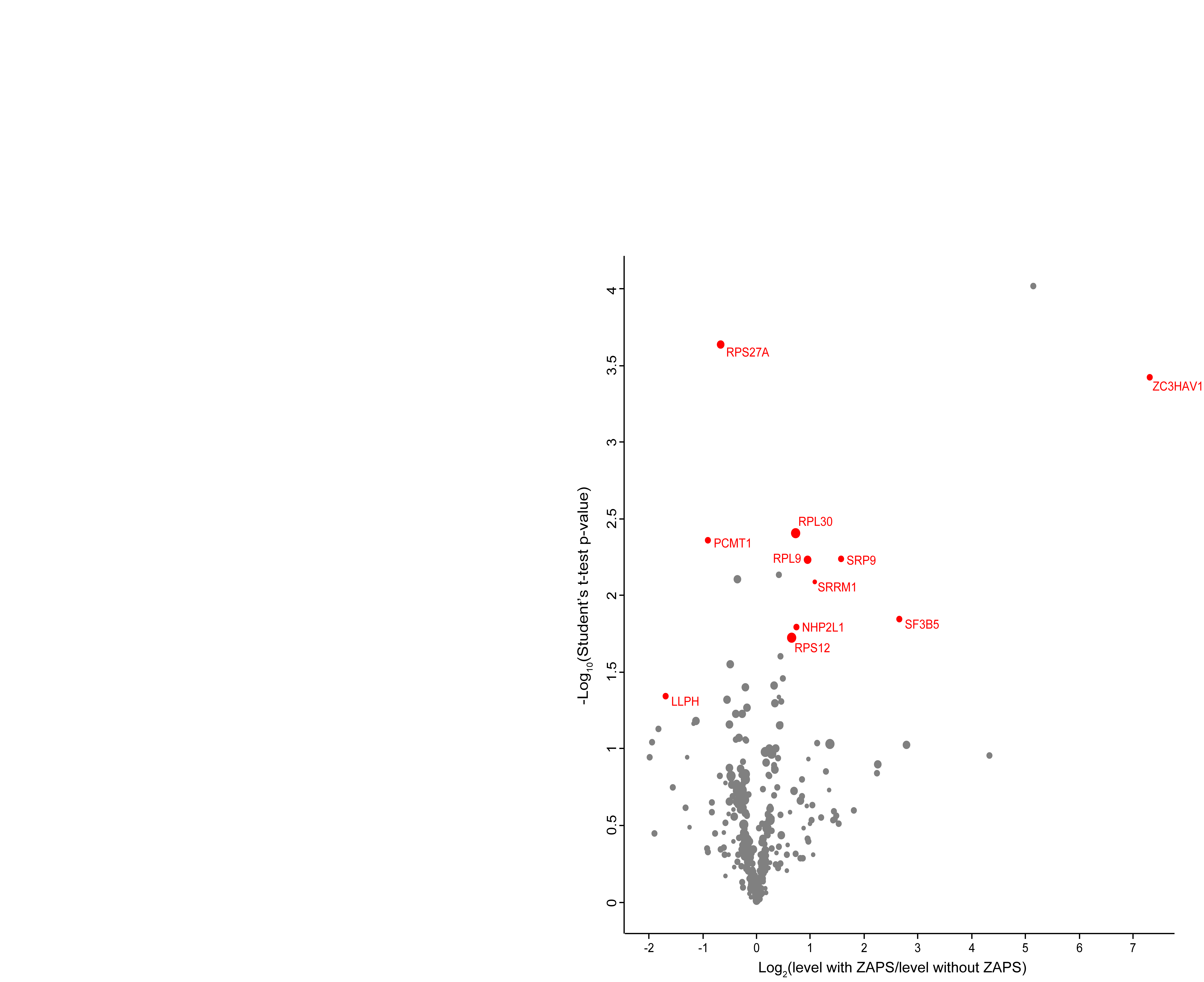

Supplement: S5 Fig — V5-tagged TRIM25 and associated proteins were co-immunoprecipitated in the absence or presence of ZAPS, and identified and quantitated by LC-MS/MS. The x-axis indicates fold differences in samples with ZAPS compared to those without (Log2) while the y-axis indicates Student’s t-test calculated p-values (-Log10); each dot represents a unique protein identified by LC-MS/MS. Red filled circles mark proteins in the TRIM25 interactome that have more than one peptide identified and a linear-fold difference of ≥1.5 (Student’s t-test p< 0.05) in the presence of ZAPS. Fold differences are calculated from LFQ values, and the size of the filled circles indicates iBAQ values for the matched proteins where the largest circles suggest the most abundant proteins (see the section on MS in Materials and Methods). (TIF) [file ppat.1006145.s005.tif]
